# Supplementary material for: Transcriptome-Based Selection and Validation of Reference Genes for Gene Expression in Goji Fruit Fly (Neoceratitis asiatica Becker) under Developmental Stages and Five Abiotic Stresses
Source: Int J Mol Sci. 2022 Dec 27;24(1):451. doi: 10.3390/ijms24010451 (PMC9820723; doi:10.3390/ijms24010451)
Supplement: Supplementary file 1 [file ijms-24-00451-s001.zip › ijms-2055992-supplementary.pdf]

Transcriptome-Based Selection and Validation of Reference Genes for Gene Expression in Goji Fruit Fly (*Neoceratitis asiatica* Becker) under Developmental Stages and Five Abiotic Stresses

Hongshuang Wei, Haili Qiao, Sai Liu, Xueqin Yuan and Changqing Xu *

Institute of Medicinal Plant Development, Chinese Academy of Medical Sciences and Peking Union Med-ical College, Beijing, 100193, China

* Correspondence: cqxu@implad.ac.cn

Table S1. Best BLAST hits for candidate reference genes and target gene identified in the transcriptome database of *N. asiatica* adults.

Gene name	transcriptome ID	Length	aa	Complete ORF	Description	E value	Identity (%)	Accession
**Reference genes:**								
α-TUB	TRINITY_DN57650_c0_g1	1731	462	Yes	tubulin alpha-4 chain [*Ceratitis capitata*]	0	95.89	XP_004526818.1
β-Actin	TRINITY_DN623_c0_g1	881	187	3' lost	actin-5, muscle-specific [*Ceratitis capitata*]	2.00E-135	99.47	XP_004518901.1
EF-1α	TRINITY_DN1467_c0_g1	4216	463	Yes	elongation factor 1-alpha 1 [*Zeugodacus cucurbitae*]	0	99.55	XP_011194365.1
EF-1β	TRINITY_DN2626_c0_g1	828	221	Yes	PREDICTED: probable elongation factor 1-beta [*Bactrocera latifrons*]	1.00E-109	92.31	XP_018798927.1
GAPDH	TRINITY_DN163_c0_g1	4234	332	Yes	glyceraldehyde-3-phosphate dehydrogenase 2 [*Ceratitis capitata*]	0	98.80	XP_004525007.1
G6PDH	TRINITY_DN1938_c0_g1	2143	496	Yes	glucose-6-phosphate 1-dehydrogenase [*Ceratitis capitata*]	0	96.98	NP_001266304.1
UBC	TRINITY_DN9782_c1_g1	1010	199	Yes	ubiquitin-conjugating enzyme E2-22 kDa [*Ceratitis capitata*]	6.00E-136	100.00	XP_004526147.1
AK	TRINITY_DN6297_c0_g1	1834	356	Yes	arginine kinase isoform X4 [*Ceratitis capitata*]	0	96.63	XP_004526799.1
GST	TRINITY_DN4702_c0_g1	1111	221	Yes	glutathione S-transferase 1 [*Ceratitis capitata*]	4.00E-122	82.81	XP_004537447.1
SDHA	TRINITY_DN3925_c0_g1	1128	116	Yes	succinate dehydrogenase assembly factor 4, mitochondrial [*Ceratitis capitata*]	2.00E-66	95.69	XP_004520046.1
TBP	TRINITY_DN3283_c1_g1	2363	325	Yes	TATA-box-binding protein isoform X1 [*Ceratitis capitata*]	0	98.77	XP_004530273.1
RPL32	TRINITY_DN4856_c0_g1	910	134	Yes	60S ribosomal protein L32 [*Ceratitis capitata*]	1.00E-93	100.00	XP_004517954.1
RPS13	TRINITY_DN9545_c0_g1	729	151	Yes	40S ribosomal protein S13 [*Ceratitis capitata*]	6.00E-105	100.00	XP_004537202.1
RPS15	TRINITY_DN1677_c0_g2	1154	130	Yes	40S ribosomal protein S15Aa [*Ceratitis capitata*]	1.00E-90	100.00	XP_004536427.1
RPS18	TRINITY_DN6053_c0_g1	1154	152	Yes	40S ribosomal protein S18 [*Ceratitis capitata*]	1.00E-89	99.24	XP_004522823.1

**Target gene:**								
OBP56a	TRINITY_DN3130_c0_g1	697	165	Yes	general odorant-binding protein 56a [*Ceratitis capitata*]	2.00E-102	87.88	XP_020718149.1

Table S2. Mean Ct values ± SE of the studied reference genes in the different samples from *N. asiatica* adults.

**Gene name**	**Odor stimulation**	**Color induction**	**Insecticide treatment**	**Starvation-refeeding**	**Temperature**	**Developmental stages**	**Sex**	**Tissues**	**All samples**
α-TUB	22.13 ± 0.58	22.12 ± 0.18	26.20 ± 0.31	24.16 ± 0.24	25.18 ± 0.10	21.35 ± 0.28	23.84 ± 0.32	24.60 ± 0.54	23.81 ± 0.22
β-Actin	18.57 ± 0.13	17.78 ± 0.16	18.38 ± 0.33	18.02 ± 0.28	18.31 ± 0.08	18.14 ± 0.32	18.50 ± 0.05	18.29 ± 0.14	18.24 ± 0.08
EF1α	17.12 ± 0.01	17.22 ± 0.01	17.58 ± 0.03	17.44 ± 0.04	17.34 ± 0.01	17.38 ± 0.01	17.34 ± 0.01	17.49 ± 0.01	17.37 ± 0.02
EF1β	28.42 ± 0.46	18.25 ± 0.10	24.32 ± 0.64	25.07 ± 0.70	21.11 ± 0.42	19.61 ± 0.21	20.20 ± 0.28	25.22 ± 1.05	22.83 ± 0.42
GAPDH	16.58 ± 0.28	15.38 ± 0.05	16.35 ± 0.04	16.78 ± 0.08	16.77 ± 0.08	16.85 ± 0.08	16.56 ± 0.10	16.50 ± 0.08	16.51 ± 0.06
G6PDH	20.60 ± 0.55	20.58 ± 0.28	24.88 ± 0.37	22.37 ± 0.25	23.85 ± 0.60	22.69 ± 0.47	22.25 ± 0.09	24.02 ± 0.75	22.89 ± 0.26
UBC	25.06 ± 0.61	21.28 ± 0.05	22.86 ± 0.61	23.88 ± 0.39	23.57 ± 0.19	22.03 ± 0.25	24.32 ± 0.24	23.46 ± 0.38	23.26 ± 0.18
AK	19.01 ± 0.43	14.84 ± 0.09	19.48 ± 0.29	18.48 ± 0.28	16.94 ± 0.31	17.48 ± 0.42	18.73 ± 0.28	17.29 ± 0.42	17.63 ± 0.19
GST	19.72 ± 0.29	17.63 ± 0.12	20.13 ± 0.06	18.64 ± 0.30	20.42 ± 0.34	19.32 ± 0.23	19.49 ± 0.05	19.63 ± 0.49	19.47 ± 0.16
SDHA	19.75 ± 0.40	20.05 ± 0.22	23.71 ± 0.13	22.41 ± 0.31	23.04 ± 0.35	21.21 ± 0.35	21.38 ± 0.17	23.11 ± 0.47	22.04 ± 0.20
TBP	24.20 ± 1.07	24.36 ± 0.53	25.50 ± 0.86	25.45 ± 0.20	27.08 ± 0.68	25.42 ± 0.33	24.71 ± 0.71	26.09 ± 0.53	25.57 ± 0.25
RPL32	18.18 ± 0.20	16.30 ± 0.17	18.06 ± 0.16	16.93 ± 0.21	20.53 ± 0.19	17.31 ± 0.25	18.20 ± 0.15	17.21 ± 0.16	17.93 ± 0.16
RPS13	16.58 ± 0.14	16.12 ± 0.05	16.99 ± 0.16	16.79 ± 0.11	16.72 ± 0.08	16.20 ± 0.04	16.83 ± 0.18	17.78 ± 0.21	16.83 ± 0.08
RPS15	28.84 ± 0.78	21.46 ± 0.45	19.72 ± 0.24	29.01 ± 0.75	19.55 ± 0.48	17.61 ± 0.25	19.31 ± 0.21	22.21 ± 0.44	21.97 ± 0.45
RPS18	17.46 ± 0.08	16.36 ± 0.07	17.65 ± 0.10	17.47 ± 0.12	17.60 ± 0.06	16.42 ± 0.05	17.23 ± 0.10	17.59 ± 0.06	17.26 ± 0.06

Table S3. Comprehensive gene stability ranking of 15 candidate reference genes in all samples was calculated using RefFinder. The Genes Geomean of ranking values is listed, and the stability decreased from top to bottom.

Rank	Odor stimulation		Color induction		Insecticide treatment		Starvation-refeeding		Temperatures		Developmental stages		Sex		Tissues		Total	
	Gene	GM	Gene	GM	Gene	GM	Gene	GM	Gene	GM	Gene	GM	Gene	GM	Gene	GM	Gene	GM
1	RPS13	2.45	EF1α	1.19	EF1α	1.00	EF1α	1.19	RPS13	2.63	RPS13	1.68	β-Actin	2.21	RPL32	3.16	RPS18	1.73
2	EF1α	2.45	RPS13	1.57	GAPDH	2.00	RPS13	2.28	RPS18	2.63	EF1α	1.86	GST	2.78	EF1α	3.31	EF1α	2.24
3	RPS18	2.66	UBC	3.13	GST	2.91	RPS18	2.38	EF1α	2.89	GAPDH	2.00	RPS13	2.91	GST	3.94	GAPDH	2.91
4	RPL32	2.99	GAPDH	3.66	RPS18	3.46	GAPDH	3.13	GAPDH	3.76	RPS18	4.24	EF1α	3.57	RPS18	4.29	RPS13	2.99
5	GST	3.20	AK	5.18	SDHA	5.23	RPL32	5.23	AK	3.95	GST	4.56	RPL32	4.41	RPS15	4.43	GST	3.46
6	GAPDH	4.90	RPS18	5.92	RPS13	5.96	TBP	5.73	GST	5.89	UBC	5.92	SDHA	4.60	GAPDH	5.05	β-Actin	5.18
7	β-Actin	6.05	GST	6.70	RPL32	6.96	α-TUB	7.00	β-Actin	5.90	EF1β	5.92	G6PDH	6.13	UBC	5.60	UBC	7.74
8	SDHA	7.48	EF1β	7.74	RPS15	7.74	G6PDH	8.24	SDHA	6.53	RPL32	8.24	UBC	7.67	AK	6.77	RPL32	8.15
9	α-TUB	9.67	β-Actin	9.24	α-TUB	9.24	GST	9.46	α-TUB	6.93	β-Actin	9.72	GAPDH	7.90	β-Actin	6.78	SDHA	8.45
10	G6PDH	10.74	α-TUB	10.24	AK	9.74	AK	10.16	UBC	8.18	SDHA	10.40	EF1β	9.74	SDHA	7.64	AK	9.46
11	UBC	10.94	RPL32	10.46	β-Actin	11.00	SDHA	10.47	RPL32	9.17	RPS15	11.06	AK	10.61	RPS13	7.93	α-TUB	11.24
12	AK	11.17	SDHA	12.00	G6PDH	12.00	β-Actin	11.93	EF1β	11.49	α-TUB	11.45	RPS18	10.82	TBP	8.11	G6PDH	11.74
13	EF1β	12.17	G6PDH	13.00	EF1β	13.49	UBC	12.49	RPS15	13.00	AK	12.18	RPS15	11.18	α-TUB	10.50	TBP	13.00
14	TBP	14.24	RPS15	14.00	UBC	13.49	EF1β	14.00	G6PDH	14.00	TBP	14.19	α-TUB	12.16	G6PDH	14.00	EF1β	14.24
15	RPS15	14.74	TBP	15.00	TBP	15.00	RPS15	15.00	TBP	15.00	G6PDH	14.24	TBP	15.00	EF1β	15.00	RPS15	14.74

Note: GM, geometric mean.

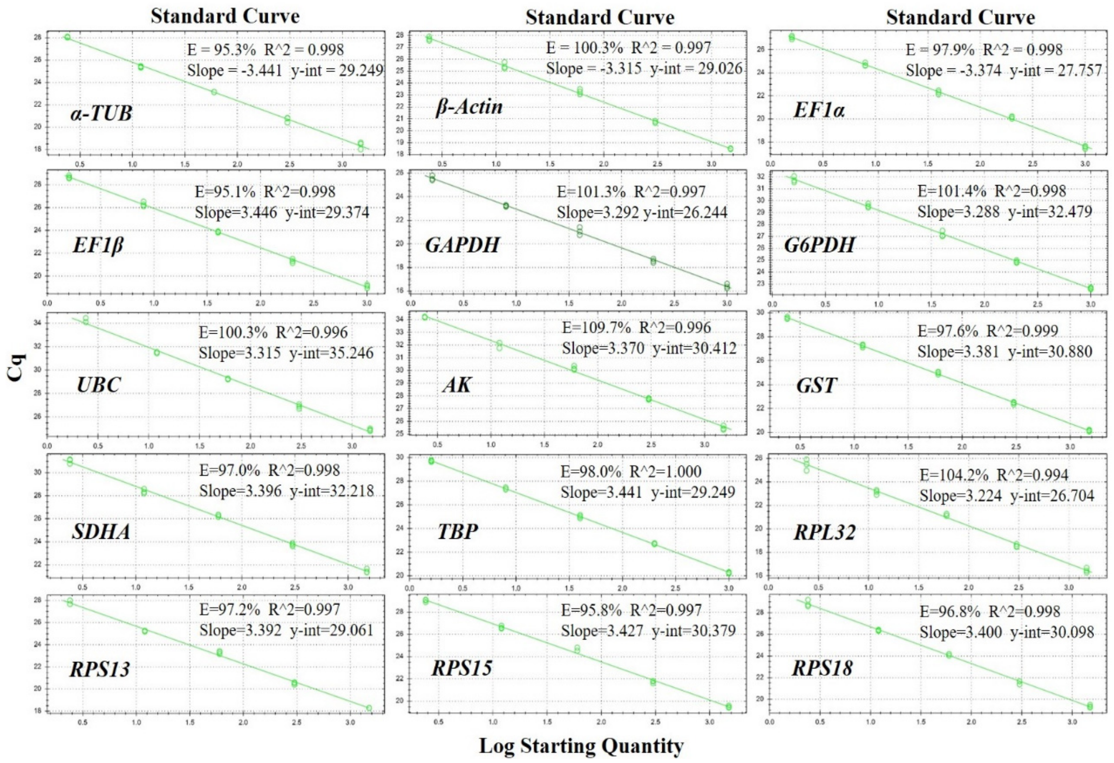

Figure S1. Efficiency of primer pairs for qPCR amplification in *N. asiatica*. The standard curves of primers for qPCR amplification of 15 candidate GRs, in-cluding *α-TUB*, *β-Actin*, *EF1α*, *EF1β*, *GAPDH*, *G6PDH*, *UBC*, *AK*, *GST*, *SDHA*, *TBP*, *RPL32*, *RPS13*, *RPS15* and *RPS18*.

```
1    ATGAAGTCCACAATCATCTGCTGCATGCTGGCTACAGCGCTACTT
      M  K  S  T  I  I  C  C  M  L  A  T  A  L  L

46   AGCTGCTCTGTACTCACCACCGAGGGACGCAAGCCAAAGAAACTA
      S  C  S  V  L  T  T  E  G  R  K  P  K  K  L

91   ACGCCGGAGCTAGAGGCGAAATTCGAGGTGCTCACCGCCTGGATT
      T  P  E  L  E  A  K  F  E  V  L  T  A  W  I

136  GCCTATCGTCTTAACCTGAAACACGCCAAGGAAGCTTGTGTTGGT
      A  Y  R  L  N  L  K  H  A  K  E  A  C  V  G

181  GAATACGGCTTTTCGGATGAACTCGCCACCAATCTGGTTAAAATT
      E  Y  G  F  S  D  E  L  A  T  N  L  V  K  I

226  AAGGTCGCCAATCCCAACGCCAATGAGAAATGTTATGTGAACTGT
      K  V  A  N  P  N  A  N  E  K  C  Y  V  N  C

271  CTCTACGATAAGCTGGTCTTTTACAAGGATAACGCCATCAATAAA
      L  Y  D  K  L  V  F  Y  K  D  N  A  I  N  K

316  CAGGCCATGAAGGAGTCTCTGTATGAAATTGTTGGTGAGCAGCGT
      Q  A  M  K  E  S  L  Y  E  I  V  G  E  Q  R

361  TTAATGGATATTGTCAATTCATGTATGAATGCTGGTGGCGCTAAT
      L  M  D  I  V  N  S  C  M  N  A  G  G  A  N

406  AACTGCGATAAGGTTTATAAGTTCCATGCGTGCGCCTCACCTCAA
      N  C  D  K  V  Y  K  F  H  A  C  A  S  P  Q

451  TTCGATAAGGTACGCGGTGATATCTTTTTGCCCGATGAATAG
      F  D  K  V  R  G  D  I  F  L  P  D  E  *
```

Figure S2. cDNA sequence and deduced amino acid sequence of *N. asiatica* OBP56a. The asterisks indicated the stop codon. The putative signal peptide region was underlined by a red double-headed arrow.

```
                                                                              identity (%)
NasiOBP56a        MKSTIICCMLATALLSCSVLTTEG---RKPKKLTPELEAKFEVLTAWIAYRLNLKHAKEA
BdorOBP56a        MKSSIICCILATVVLSLCVFNADAGL-RKPKKLTPELEAKFEVLTAWIAYRLNLKHAKEA    81.18
BminOBP56a        MKSSIICCILATVLLSLCTFGTDAALGRKPKKLTPELESKFEVLTAWIAYRLNLKHAKEA    89.80
BtryOBP56a        MKSSIICCILATVLLSLCVFNAEAGL-RKPKKLTPELEAKFEVLTAWIAYRLNLKHAKEA    82.35
RpomOBP56a        MKSTTSYCILVAVLFCLCVSNTEAFG-RKAKKLNPELEAKFDVLTAWIAYRLNLKHAKEA    73.53
ZcucOBP56a        MKYSIICCMLATVLISLCVFSADAGL-RKPKKLTPELEAKFEVLTAWIAYRLNLKHAKEA    81.76
                  **     * *                 ** *** **** ** ******************

NasiOBP56a        CVGEYGFSDELATNLVKIKVANPNANEKCYVNCLYDKLVFYKDN-AINKQAMKESLYEIV
BdorOBP56a        CVGEYGFSDELATNLVKIKVANPSDREKCYVNCLYNKLVFYKDD-AINKQAMKESLYEIV
BminOBP56a        CVGEYGFSDELATNLVKIRVANPSDNEKCYVNCLYNKLVFYKDN-AINKQAMKESLYEIV
BtryOBP56a        CVGEYGFSDELATNLVKIKVANPNDREKCYVNCLYNKLVFYKDD-AINKQAMKESLYEIV
RpomOBP56a        CIGEYGFTDELATSLVKVQVANPSDREKCYVNCLYTKLVFYNNN-QINTQAMKESLIEIV
ZcucOBP56a        CVGEYGYSDELATNLVKIKVANPTDQQKCYVNCLYNKLVFYKDD-AINKQAMKESLYEIV
                  * ****  ***** ***  ****    ******** *****     ** ******* ***
                  C1                          C2  C3

NasiOBP56a        GE---QRLMDIVNSCMNAGGANNCDKVYKFHACASPQFDKVRGDIFLPDE
BdorOBP56a        GE---QRLLNIVDGCLNAGGTNACDKVYKFHACASPEFDKVRSDIFLPDE
BminOBP56a        GE---QRLMNIVNGCLNAGGTNACDKVYKFHACASPEFDKVRSDIFLPDE
BtryOBP56a        GE---QRLLNIVDGCLNAGGTDACDKVYKFHACASPEFDKVRSDIFLPDE
RpomOBP56a        GE---ERLLQIVNSCLNAGGANDCDKVYKFHACASPEFDKVRGDIFQPDE
ZcucOBP56a        GE---QRLMNIVNGCLNAGGTNACDKVYKFHACASPEFDKVRGDIFLPDE
                  **    **  **  * ****   ************* ***** *** ***
                              C4       C5        C6
```

Figure S3. Comparison of the amino acid sequence of the NasiOBP56a with those of other species. Identical amino acid residues in all proteins are indicated by asterisks. The conserved six cysteine residue in sequence were marked in red. The blue numbers indicated the comparison of similarity of the protein sequences between NasiOBP56a and those of other species. BdorOBP56a: *Bactrocera dorsalis* OBP56a (GenBank accession number: AKI29008.1). BminOBP56a: *Bactrocera minax* OBP56a (acc. number: AYN70638.1). BtryOBP56a: *Bactrocera tryoni* OBP56a (acc. number: XP_039955300.1). RpomOBP56a: *Rhagoletis pomonella* OBP56a (acc. number: XP_036333622.1). ZcucOBP56a: *Zeugodacus cucurbitae* OBP56a (acc. number: JAC98783.1).

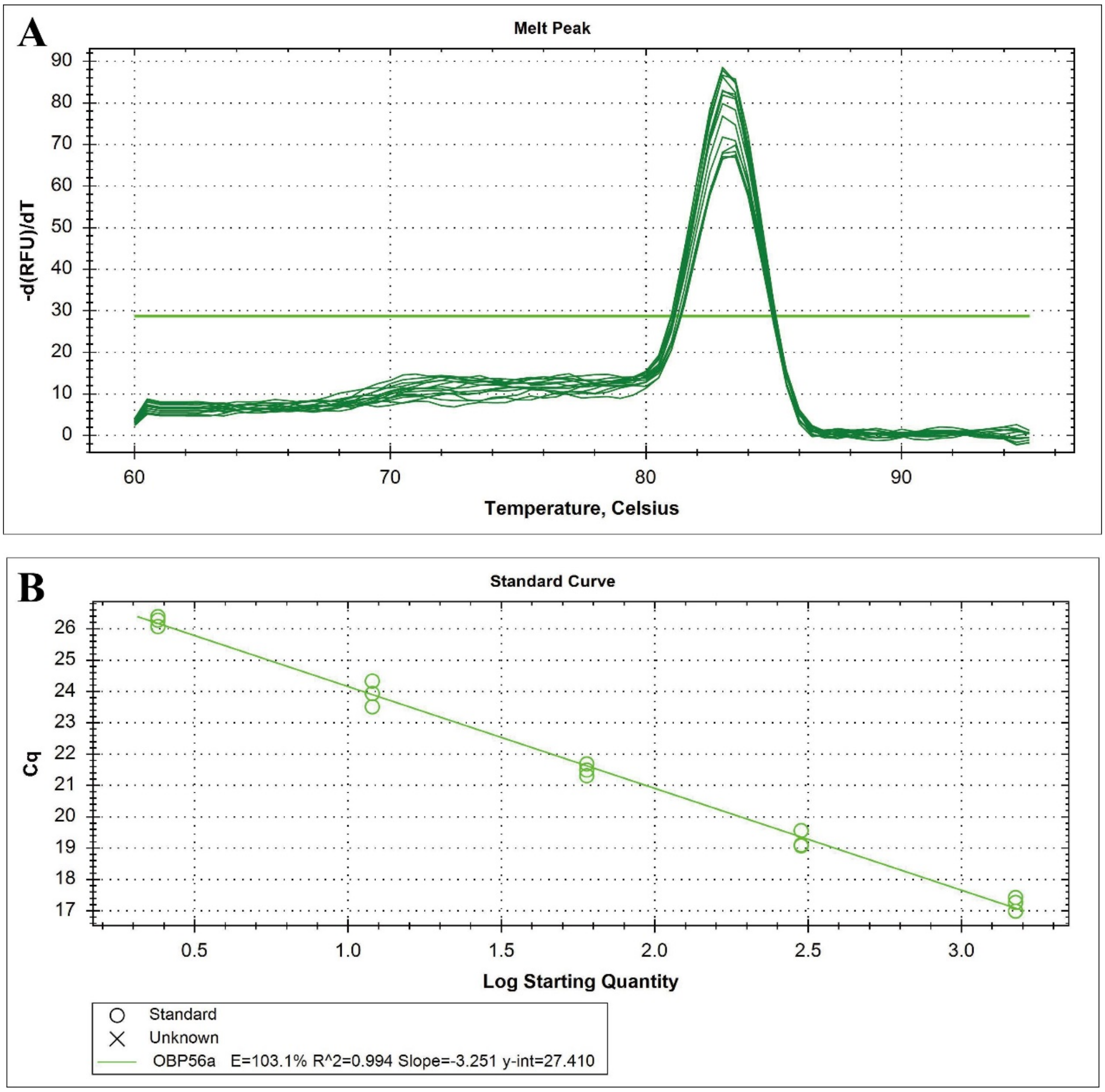

Figure S4. Specificity and efficiency of primer pairs of target gene for RT-qPCR amplification in *N. asiatica.* A: The melt peaks and standard curves of primers for RT-qPCR amplification of target gene *OBP56a*. B: The standard curves of primers for RT-qPCR amplification of target gene *OBP56a*.
